# Supplementary material for: Potential for high dynamic range Sedia Limiting Antigen antibody assay to support viral load monitoring during antiretroviral therapy
Source: PLoS One. 2024 Jun 6;19(6):e0303393. doi: 10.1371/journal.pone.0303393 (PMC11156293; doi:10.1371/journal.pone.0303393)
Supplement: S1 Table — (DOCX) [file pone.0303393.s001.docx]

**Supplementary File 1**

**S1 Table 1: Multivariate linear regression among patients who were suppressed throughout follow-up (category two) and those who had at least one detectable viral load (Category three)**

| Characteristic | Patients who had fully suppressed (<1000copies/mL) throughout ART follow-up | | | Patients with a detectable viral load | | |
| --- | --- | --- | --- | --- | --- | --- |
|  | **Beta** | **95% CI** | **p-value** | **Beta** | **95% CI** | **p-value** |
| Sex |  |  |  |  |  |  |
| F | — | — |  | — | — |  |
| M | 0.00 | 0.00, 0.00 | >0.9 | 0.00 | -0.01, 0.01 | 0.9 |
| Age in years | 0.00 | 0.00, 0.00 | 0.9 | 0.00 | 0.00, 0.00 | 0.2 |
| HIV subtype |  |  |  |  |  |  |
| B | — | — |  | — | — |  |
| C | 0.00 | -0.01, 0.00 | 0.9 | 0.00 | -0.02, 0.01 | 0.5 |
| Other | 0.00 | 0.00, 0.00 | 0.6 | 0.00 | -0.02, 0.01 | 0.8 |
